# Magneto-nanosensor platform for probing low-affinity protein–protein interactions and identification of a low-affinity PD-L1/PD-L2 interaction

**DOI:** 10.1038/ncomms12220

**Published:** 2016-07-22

**Authors:** Jung-Rok Lee, Daniel J. B. Bechstein, Chin Chun Ooi, Ashka Patel, Richard S. Gaster, Elaine Ng, Lino C. Gonzalez, Shan X. Wang

**Affiliations:** 1 Department of Mechanical Engineering, Stanford University, Stanford, California 94305, USA; 2 Department of Chemical Engineering, Stanford University, Stanford, California 94305, USA; 3 Department of Proteomics and Biological Resources, Genentech, South San Francisco, California 94080, USA; 4 Department of Bioengineering, Stanford University, Stanford, California 94305, USA; 5 Division of Plastic and Reconstructive Surgery, Harvard University, Boston, Massachusetts 02114, USA; 6 Department of Materials Science and Engineering, Stanford University, Stanford, California 94305, USA; 7 Department of Electrical Engineering, Stanford University, Stanford, California 94305, USA

## Abstract

Substantial efforts have been made to understand the interactions between immune checkpoint receptors and their ligands targeted in immunotherapies against cancer. To carefully characterize the complete network of interactions involved and the binding affinities between their extracellular domains, an improved kinetic assay is needed to overcome limitations with surface plasmon resonance (SPR). Here, we present a magneto-nanosensor platform integrated with a microfluidic chip that allows measurement of dissociation constants in the micromolar-range. High-density conjugation of magnetic nanoparticles with prey proteins allows multivalent receptor interactions with sensor-immobilized bait proteins, more closely mimicking natural-receptor clustering on cells. The platform has advantages over traditional SPR in terms of insensitivity of signal responses to pH and salinity, less consumption of proteins and better sensitivities. Using this platform, we characterized the binding affinities of the PD-1—PD-L1/PD-L2 co-inhibitory receptor system, and discovered an unexpected interaction between the two known PD-1 ligands, PD-L1 and PD-L2.

## Abstract (web summary)

Measurement of low-affinity protein–protein interactions is challenging; surface plasmon resonance requires high concentrations of reagents. Here the authors combine magneto-nanosensors with microfluidic chips and protein-conjugated magnetic nanoparticles to discover a low-affinity interaction between T-cell inhibitory receptors PD-L1 and PD-L2.

## Graphical abstract

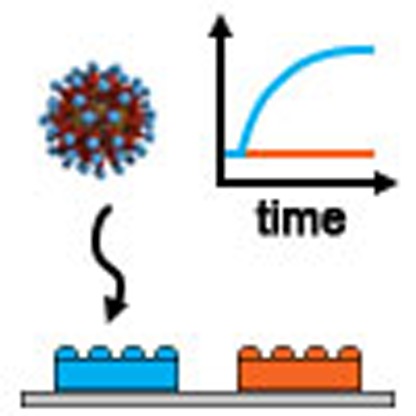

It has been widely appreciated that targeting genome sequences, key proteins and pathways by new immunomodulatory treatments are fertile grounds for drug development^1^. For example, leukocyte cell-surface receptors interact with tumour cells and tumour environment, and thus are attractive targets for immunotherapies^2^. Considerable efforts have been made to understand the interactions and their functions via these cell-surface molecules such as the T-cell receptor^3^,^4^ and costimulatory receptor CD28 (refs 5, 6). Recently, among the cell-surface molecules, inhibitory receptors (also known as immune checkpoint receptors) have been extensively studied in cancer to enhance T-cell-mediated antitumour response^7^,^8^,^9^. The best-studied inhibitory receptors, cytotoxic T lymphocyte-associated antigen-4 (refs 10, 11) and programmed cell death protein 1 (PD-1)^12^,^13^, have even led to immunotherapies that have achieved US Food and Drug Administration (FDA) approval and translation to the clinic.

In immune checkpoint therapy, where blocking inhibitory receptor–ligand interactions enhances antitumour responses, a fundamental understanding of the interactions between inhibitory receptors and their ligands is crucial to elucidate the mechanism of action. Critical elements are the identification of all interacting partners and the measurement of binding affinities between their extracellular domains. A challenge, however, is that dissociation constants of known interactions between leukocyte cell-surface molecules, as measured by surface plasmon resonance (SPR)^14^, can range from a few to several hundred micromolars (μM)^15^. To measure such a low-affinity interaction with SPR, the current gold standard^16^,^17^, high concentrations of reagents are required up to a comparable level of its dissociation constant or even a few orders of magnitude higher, which in some cases may be impractical with regards to protein solubility and expense. To address these issues, magneto-nanosensors with better sensitivities have been developed to perform kinetic binding measurements^18^. However, due to binding signals being coupled with diffusion rates in a stationary solution, our prior work on magneto-nanosensors relied heavily on a two-compartment model to estimate kinetic parameters.

Here, we present a much-improved platform where magneto-nanosensors are integrated with microfluidic chips to measure the dissociation constants of low-affinity interactions in a multiplex manner by flowing protein-conjugated magnetic nanoparticles (MNPs) into microchannels over magneto-nanosensors coated with binding or non-binding partners. Conjugated MNPs at the surface are replenished by a continuous flow enabled by the microfluidic chips, reducing a previously-derived two-compartment binding model^18^ with a simple Langmuir isotherm. We then utilize this magneto-nanosensor platform to estimate the affinities of interactions between PD-1 (CD279), its ligands PD-L1 (B7-H1 or CD274) and PD-L2 (B7-DC or CD273), and B7-1 (CD80). Interestingly, the improved platform facilitates the discovery of a new interaction between PD-L1 and PD-L2, which we subsequently confirm using an unbiased cell-based, receptor-interaction screen.

## Results

### Magneto-nanosensor platform and MNP tags

**Figure 1:**
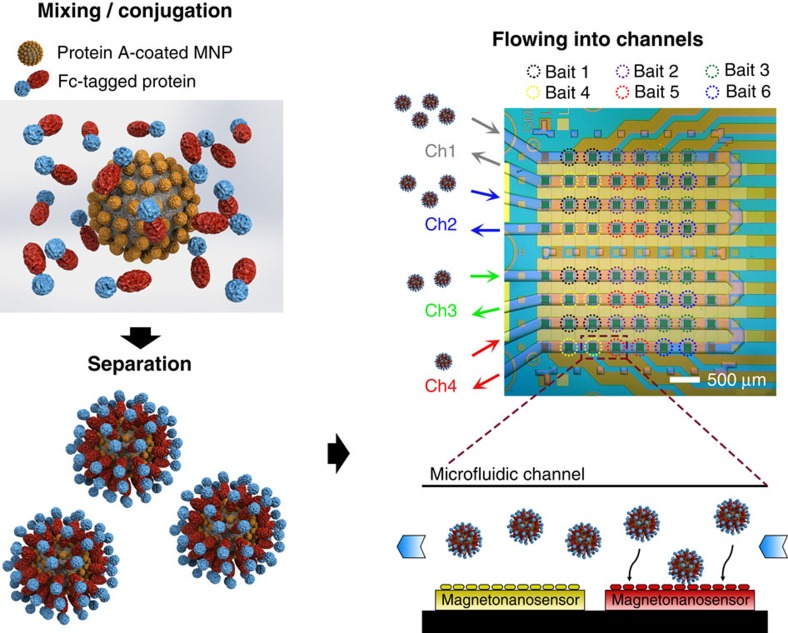
Schematic of kinetic assay with magneto-nanosensor platform. Protein A-coated MNPs were conjugated with Fc-tagged proteins. The complexes were separated from the mixture, and serial dilutions of the complexes were flowed into four microfluidic channels where six different baits were immobilized on the sensors in duplicate. The dimension of each magneto-nanosensor is 100 × 100 μm, and the microfluidic channel width is 200 μm. The solutions containing the complexes were delivered by syringe pumps individually connected to each channel.

The magneto-nanosensor platform is based on a magneto-nanosensor chip integrated with a microfluidic chip to perform a kinetic assay with MNP complexes. A magneto-nanosensor chip is an array of magnetic sensors that can detect MNPs in their proximity, employing the effect of giant magnetoresistance (GMR). It has been mainly used to measure protein biomarkers in immunoassay formats^19^,^20^,^21^ after the advent of the initial concept^22^. For kinetic assays with the magneto-nanosensors, proteins of interest (prey) need to be pre-conjugated with MNPs instead of being sequentially added as in the immunoassays. Upon binding of the prey-MNP complexes to the proteins on the sensors (bait), the magneto-nanosensors produce signals proportional to the number of bound complexes^18^,^23^. To conjugate MNPs with prey proteins, Fc-tagged proteins and MNPs coated with protein A from *Staphylococcus aureus* were mixed to link them via the interaction between protein A and the Fc-region (Fig. 1). To saturate all protein A's on the surface of the MNP, a 1,000 times higher concentration of Fc-tagged proteins compared with the concentration of the MNPs was incubated with the MNPs. Unbound Fc-tagged proteins were washed away using magnetic columns that can retain the conjugated MNPs under an external magnetic field. The conjugated MNPs were eluted from the column after removing the magnetic field. Bait proteins, including both binding and non-binding partners, were immobilized on the sensors in duplicates and used to test interactions with prey proteins. Since the bait proteins that we used are also Fc-tagged, an additional blocking step with soluble protein A have been conducted before the complexes were flowed in to avoid any undesirable protein A-mediated bindings, as utilized previously^24^. For immobilization of proteins on the sensors, proteins at 1 mg ml^−1^ were deposited to fully cover the surface, allowing bivalent interactions to take place with multiple prey proteins on the MNP. Due to flexibility of linkage and geometry, only a few prey proteins on the MNP are directly involved with the interaction^25^. Furthermore, other studies showed that nanoparticles (with comparable size to ours) with multivalency (more than four) have a converged dissociation constant over various polyvalencies^26^,^27^, and it has been suggested that the bivalent mode is predominant in the interaction^27^. In our case, the MNPs have a capacity of conjugation with at least 150 prey proteins (Supplementary Fig. 1). If we assumed either monovalent or trivalent mode of binding, the resulting kinetic calculations would have yielded substantial discrepancy in estimated dissociation constants (Supplementary Table 1). Thus, it is reasonable to conclude that the bivalent mode is the main contribution of binding in these interactions. A four-channel microfluidic chip made of polydimethylsiloxane (PDMS) was aligned on the magneto-nanosensor chip with an 8 × 8 array of sensors to allocate 16 sensors per channel (Fig. 1). For the experiments described in this paper, a multi-channel syringe pump was used to flow different types of solutions or serially diluted solutions containing MNPs into the channels individually. The immobilization pattern of bait proteins and the direction of flow were identical across the channels (Supplementary Fig. 2).

### Measurement of real-time binding curves

**Figure 2:**
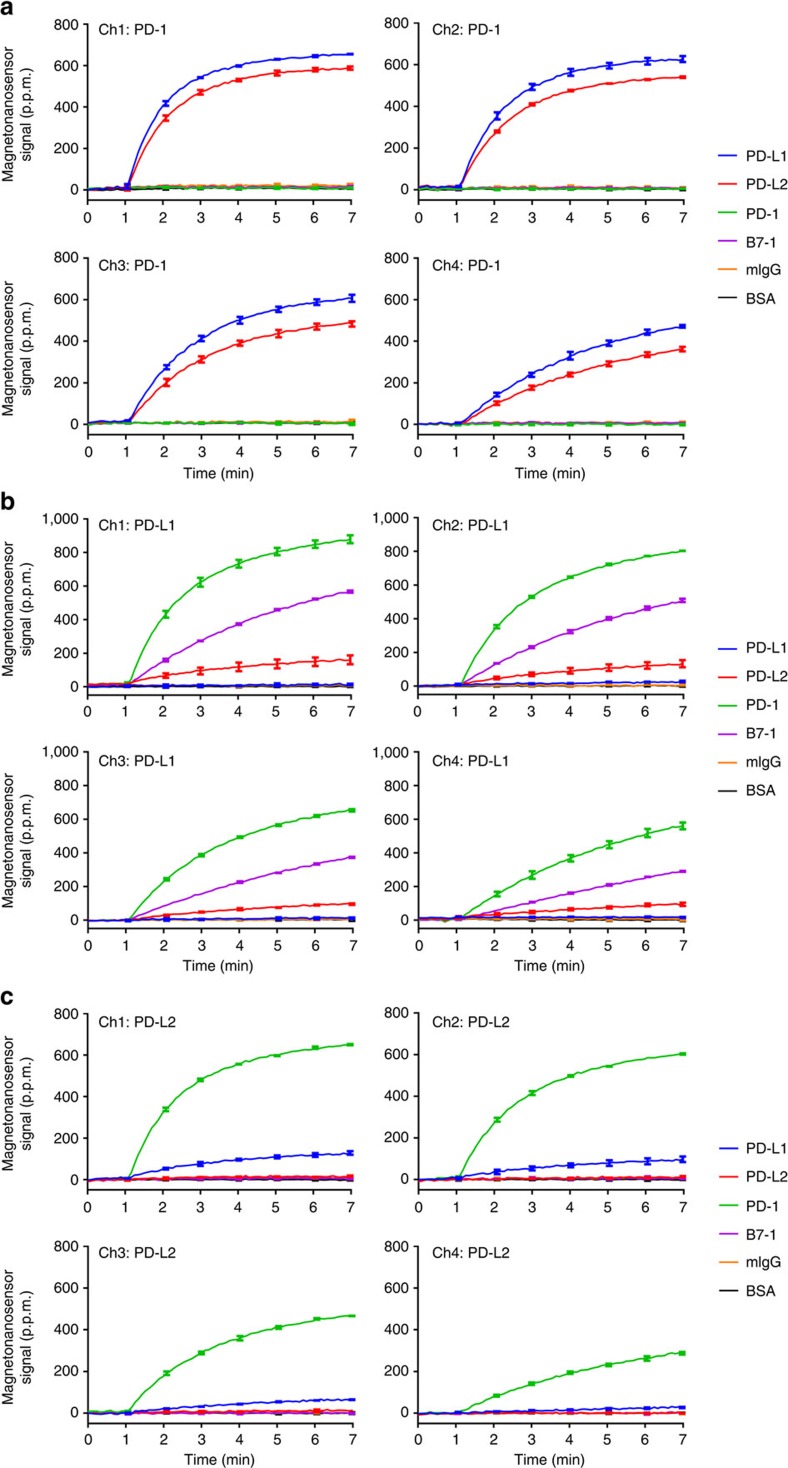
Use of magneto-nanosensor platform to measure binding curves of human PD-1 systems. Binding curves of (**a**) PD-1 complexes, (**b**) PD-L1 complexes and (**c**) PD-L2 complexes to bait proteins on the sensors. Different colours indicate the binding curve for specific bait proteins (blue: PD-L1, red: PD-L2, green: PD-1, purple: B7-1, orange: murine IgG and black: BSA). The highest concentration (100% of eluted concentration of the complexes) was flowed through channel 1 (Ch1), and the serially diluted complexes (75, 50 and 25% of the eluted concentration of the complexes) were separately flowed through channel 2 (Ch2), channel 3 (Ch3) and channel 4 (Ch4), respectively. The binding curves were post-synchronized across the channels to align the onsets at 1 min, and the error bars represent standard deviations of signals from two identical sensors.

To demonstrate the ability of our magneto-nanosensor platform to detect micromolar affinity interactions, human PD-1 and its ligands were tested (Fig. 2). After MNPs were conjugated with prey proteins (one of PD-1, PD-L1, PD-L2 and B7-1), the complexes were eluted and serially diluted to be 100 (undiluted), 75, 50 and 25%, respectively, of the eluted concentration. These four different concentrations of the same complexes were flowed through different channels, respectively, at 1 μl min^−1^. Each channel had the same immobilization pattern of bait proteins on the sensors. PD-1, PD-L1, PD-L2 and B7-1, which were used as prey proteins conjugated with MNPs (as just described), were also immobilized as bait proteins on the sensors, along with two negative controls such as murine IgG and bovine serum albumin (BSA).

When the complexes with PD-1 were flowed through the channels, the binding signals were observed only from both PD-L1 and PD-L2 immobilized sensors, which are known ligands of PD-1 (Fig. 2a). PD-1 did not interact with other non-binding partners or itself, and the signals from them remained low and constant during the course of measurement. As the concentration decreased across channels (highest in channel 1 and lowest in channel 4), the binding rates were reduced. Similarly, when the complexes with PD-L1 were flowed over another chip, they bound to PD-1 and its recently discovered receptor^28^, B7-1, on the sensors (Fig. 2b). Surprisingly, a novel interaction between PD-L1 and PD-L2 was discovered. This interaction was again observed when PD-L2 complexes were flowed over PD-L1 immobilized sensors, switching the orientation of the proteins (Fig. 2c).

**Figure 3:**
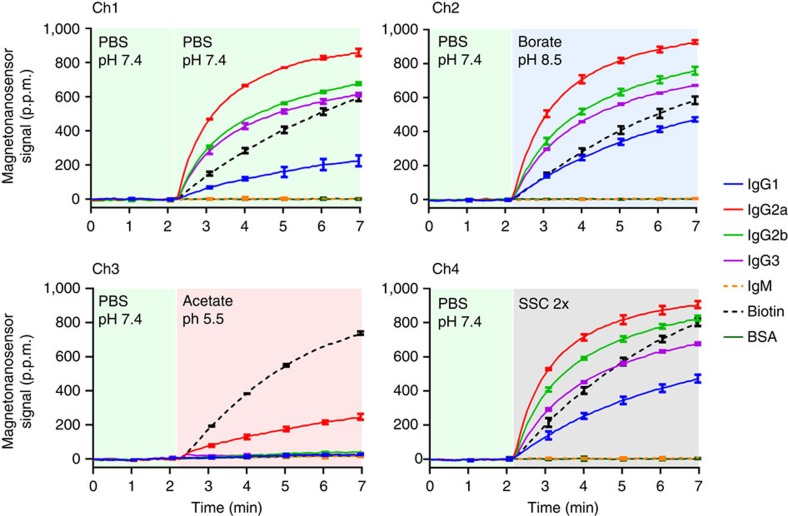
Insensitivity of magneto-nanosensors to pH and salinity. A mixture of protein A-coated and streptavidin-coated MNPs at different pH (PBS: pH 7.4, borate: pH 8.5, acetate: pH 5.5) and salt concentrations (SSC 2 × : 0.3 M NaCl) were flowed into the four channels instead of the protein complexes. The sensors in each channel were identically functionalized with murine IgG isotypes (IgG1, IgG2a, IgG2b and IgG3), murine IgM, biotin (biotinylated BSA) and BSA. The binding signals were the average signal of two identical sensors with the same protein immobilized, and the binding curves were post-synchronized across the channels. The error bars represent standard deviations of signals from the two identical sensors.

Notably, the baseline signal of the magneto-nanosensor was not shifted, unlike SPR, when the samples were introduced at around 1 min (Fig. 2). To further test the sensitivity of magneto-nanosensors to pH and salinity, different types of solutions containing the same amounts of both protein A-coated and streptavidin-coated MNPs were flowed into different channels where murine IgG isotypes, IgM, BSA and biotinylated BSA were immobilized, respectively (Fig. 3). Initially, all channels were filled with phosphate-buffered saline (PBS) at pH 7.4, and MNP solutions at the same or different pH and at a higher salt concentration were introduced. During the transient period, the signals from non-binding partners (negative controls: IgM and BSA) remained constant, which indicates that the magneto-nanosensor signals are insensitive to changes in pH. At pH 5.5, the interactions between protein A and all murine IgG isotypes except IgG2a were impaired, which is a well-known phenomenon^29^. In contrast, the binding between biotin and streptavidin was stable within the pH range^30^. In addition, salinity did not significantly affect the magneto-nanosensor signals from these interactions, and no stepwise change was generated in the signals when the high salt solution, SSC 2 × (saline sodium citrate 2 × : 0.3 M NaCl), was introduced into the channel (Fig. 3). Under similar conditions, baseline shifts or stepwise changes would be easily observed in SPR signals^14^,^31^. Importantly, when the same concentrations of protein A-coated MNPs were flowed into two identical channels where murine IgG isotypes were immobilized, the binding curves from the channels were highly reproducible (Supplementary Fig. 3), attesting to the robustness of the magneto-nanosensor and the new developed kinetic assay protocol.

### Calculation of kinetic parameters

Since the flow rate of the complexes was sufficiently fast to reach the reaction-limited regime (Supplementary Note 1), the binding kinetics can be expressed simply by the Langmuir isotherm as equation (1), and is mainly determined by the concentration of the complexes:


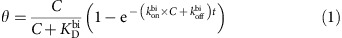


where *θ* is the coverage of bait proteins bound to the complexes, *C* is the concentration of the complexes in solution, 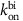 and 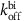 are the association and dissociation rate of the bivalent interaction, respectively, and the dissociation constant of the bivalent interaction, 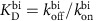. The kinetic parameters were denoted with ‘bi' because the measurement involved bivalent interactions. Since the magneto-nanosensor signals are proportional to the coverage of MNPs^23^, the binding signals obtained with the magneto-nanosensors also follow equation (1).

**Figure 4:**
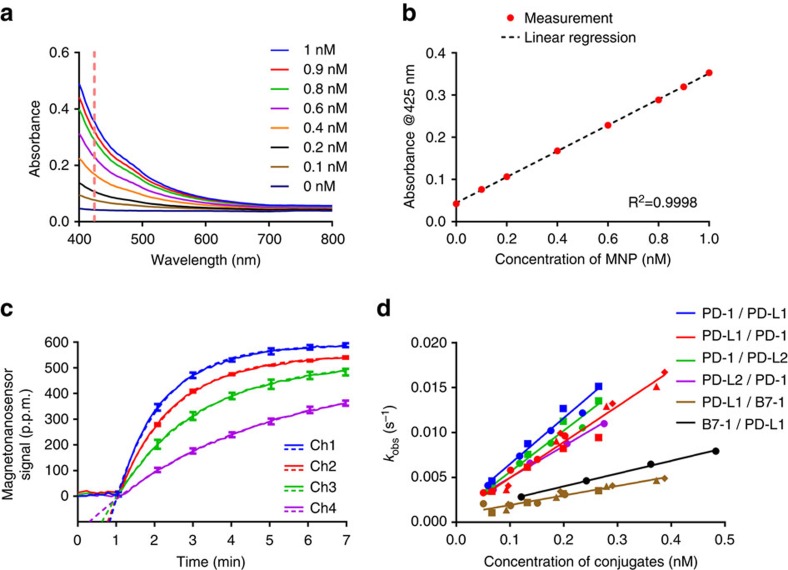
Calculations of MNP concentrations and kinetic parameters. (**a**) Absorbance measurement versus the concentration of MNPs. The MNPs at known concentrations were scanned from 400 to 800 nm. (**b**) Calibration curve (absorbance at 425 nm versus corresponding concentrations). (**c**) An example of fitting curves. Binding curves of PD-1 complexes to PD-L2 at different concentrations were plotted with their fitting curves. The solid curve is the binding signal, and the dashed curve is the fitting curve. The error bars represent standard deviations of binding signals from two identical sensors. (**d**) The observed rates versus corresponding concentrations. Different symbols indicate different sets of experiments performed on different days. The first protein listed in the legend is the prey and the second protein is the bait.

To determine the concentration of complexes, the absorbance of MNPs at different known concentrations was measured from 400 to 800 nm (Fig. 4a). Since the results showed that MNPs absorb blue light, a wavelength of 425 nm was selected to establish a calibration curve (Fig. 4b). The relationship between absorbance and the corresponding concentration was highly linear. This calibration curve was used to determine the concentrations of complexes in subsequent analyses for the kinetic parameters.

Using non-linear regression, the binding curves were fitted with a simple exponential function shown as equation (2) (Fig. 4c):


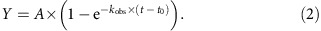


The binding signals and fitting curves were significantly well-matched with only two variables: the amplitude, *A*, and the observed binding rate, *k*_obs_ (excluding time shift, *t*_0_), which indicates that the flow rate was fast enough to replenish the complexes with minimal depletion and hence, the simplified binding model is valid. Since the apparent binding rate (*k*_obs_) is coupled with 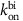 and 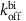, an additional analysis is required to decouple the parameters without directly measuring 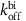 alone by flowing buffer solution and capturing the dissociation curve. In addition, due to the bivalent interaction, the dissociation rate has been reduced so it is difficult to monitor significant signal drops induced by unbinding events within a practically reasonable time period.

The exponent (*k*_obs_) of the exponential function has a simple relationship with 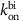 and 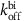 of the interaction, which is 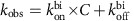, where *C* is the concentration of the complexes. If the exponents are plotted versus corresponding concentrations as determined by absorbance, the slope and the *y*-intercept are 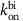 and 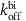 of the bivalent interaction, respectively (Fig. 4d). The calculated 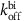 agreed well with the value obtained from the direct measurement of dissociation curve (Supplementary Fig. 4). The values of 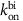 and 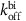 for the various interactions were subsequently obtained, and the dissociation constant of the monovalent interaction was calculated (Table 1), using equation (3)^32^:


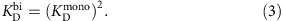


### The interaction of PD-L1 and PD-L2

**Figure 5:**
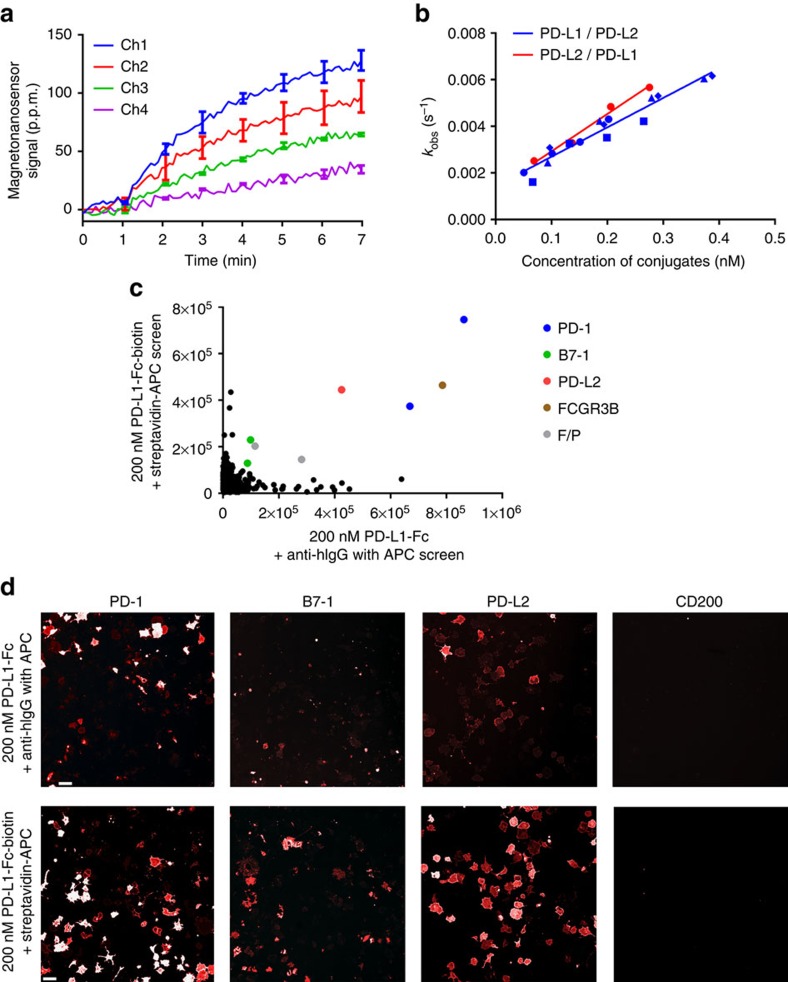
Novel interaction. (**a**) Binding curve of PD-L2 complexes at different concentrations to immobilized PD-L1 in different channels. The error bars represent s.d. of two identical sensor signals. (**b**) The observed rates plotted versus the corresponding concentrations. Different symbols with the same colour indicate the same type of experiments performed on different days. The first protein listed in the legend was the prey, and the second protein was the bait. (**c**) Scatter plot representing data from expression-cloning screens of a full-length human single-transmembrane protein library against PD-L1–Fc and PD-L1–biotin–streptavidin complex. Relevant hits are coloured blue (PD-1), green (B7-1) and red (PD-L2), respectively. The screens also recognized FCGR3B, which is a low-affinity Fc receptor, and two false positives (F/P). These false positives were due to artifacts evident upon image inspection. (**d**) Immunofluorescence images of hit wells showing cell-surface bindings on transfected COS-7 cells. Scale bars, 100 μm. The images of CD200-expressing cells are shown as a representative of negative controls.

To our knowledge, the interaction between PD-L1 and PD-L2 has not been described in the literature. The binding curves of PD-L2 complexes to PD-L1 on the sensor surface showed a dose response, indicative of a true protein–protein interaction (Fig. 5a). One possibility for why this interaction has not been identified before is that the binding affinities might be exceptionally low. To evaluate this possibility, the kinetic parameters were calculated as described above (Fig. 5b). Interestingly, the dissociation constants of the monovalent interaction were calculated to be 10.7 μM (PD-L1 flowed) and 9.1 μM (PD-L2 flowed), showing that their affinities are not significantly lower than other interactions in this receptor-interaction network. In addition, the interaction appears to be human-specific, because the interaction between murine PD-L1 and PD-L2 was not detected using our platform (Supplementary Fig. 5).

To determine if the interaction was detectable using SPR, we conducted a series of Biacore binding experiments between PD-L1 and PD-L2, and controls from 0.5 to 10 μM analyte concentrations. However, we were unable to detect any binding response using SPR (Supplementary Fig. 6).

Next, to evaluate if the PD-L1/PD-L2 interaction could be identified using a cell-based binding assay^33^,^34^ instead of planar assays, we carried out a qualitative unbiased expression-cloning screen, using an extensive single-transmembrane (STM) receptor cDNA library (∼1,900 cDNA clones, representing 90% of predicted human STM proteins). To compensate for low-affinity interactions, PD-L1 was screened both as an Fc-fusion and as a multimeric biotin–streptavidin complex. Interestingly, the known interaction between PD-L1 and B7-1 showed a relatively low-signal response (Fig. 5c). This is consistent with our own observations that the PD-L1/B7-1 interaction is difficult to detect on the cell surface (unpublished results). In both datasets, however, there are clear hits against the two clones of PD-1 and a single clone of PD-L2 present in the library (Fig. 5c). These results confirm the binding activity between PD-L1 and PD-L2 in the context of the cell membrane (Fig. 5d).

## Discussion

The key aspect of the magneto-nanosensor platform is to utilize bivalent interactions, which enables the measurement of micromolar-ranged interactions with less reagent consumption. Combined with this, the multiplexing capabilities of the platform further reduced the total amount of reagents for multiple tests. Especially for screening low-affinity interactions, it is considerably expensive to supply the reagents required for the tests. Another important advantage of the multiplexing capability is that it enables high-throughput kinetic assays. This dose not only reduce the total assay time, but also allows highly precise comparison between multiple binding partners against the same prey protein because the binding partners on different sensors are exposed to exactly the same solution containing the prey proteins under the same condition. In other words, we can minimize the risks of experimental variations between multiple measurements with the identical condition performed separately. Integration with microfluidic chips simplifies the mathematical binding model described in the previous work^18^, and compartmentalizes the sensors to perform multiple tests with different concentrations of prey proteins in parallel. Incorporated with the binding rate measurement, this allows removing the need for regeneration of the chip, which is inevitably performed in SPR to obtain a set of data with different concentrations of prey proteins. Thus, the efforts to find the optimal regeneration condition to retain activity of bait proteins over multiple cycles of tests with different concentrations of prey proteins are no longer needed in the magneto-nanosensor platform. In addition, SPR measures the changes in refractive index induced upon binding of prey proteins in solution to bait proteins immobilized on the surface. The refractive index, however, is also affected by changes in pH or salt concentration of the solution, which inevitably results from switching solutions between samples and buffers. These unpredictable and undesirable changes in signal introduce additional complexity into the analysis. Insensitivity of our platform to pH and salinity removes the need for a complicated algorithm to decouple pure binding signals from obtained sensor signals. This feature also provides the ability to study the dependency of protein–protein interactions on pH or salinity, which was clearly demonstrated in this paper.

Our new platform for kinetic assays can be readily scaled up. The number of data points obtained with a single magneto-nanosensor chip is currently limited by the number of sensors and microfluidic channels per chip. Since the magneto-nanosensor chips are easily scalable (for example, from 64 sensors up to 256 sensors) with minimal loss in performance^35^, a quadruple number of data points can be obtained at a time to reduce measurement variations and fitting errors compared with the current setting. Alternatively, more prey proteins, bait proteins or binding conditions can be interrogated in a single 256-sensor chip. To accommodate even more advanced kinetic studies, we have found a path forward for realizing magneto-nanosensor chips with thousands of sensors^36^.

The novel interaction between PD-L1 and PD-L2 was detected with two independent modalities: magneto-nanosensor platforms and cell-based expression-cloning screens. This interaction, however, was not observed with SPR, which may be a reason that it has not been discovered until now. It is possible that immobilization of bait proteins on the SPR sensor surface masks the PD-L1/PD-L2 binding epitope, but not PD-1 or B7-1 binding surface. The different sensor surface used here may allow access to a fraction of the immobilized molecules on the magneto-nanosensor.

Although the functional implications of the novel interaction between PD-L1 and PD-L2 in immune response and cancer checkpoint activity remain to be determined, both of these PD-1 ligands can in some circumstances be co-expressed by tumour cells, as exemplified by expression studies on Reed–Sternberg cells in Hodgkin's lymphomas^37^. In this disease, both PD-L1 and PD-L2 are common targets of chromosome 9p24.1 amplification^38^. It will be important to determine if the PD-L1/PD-L2 ligands are capable of interacting in *cis*- (on the same cell surface) or *trans*-orientations (between cells). Our binding experiments, using GMR and expression cloning, do not necessarily define the orientation of the potentially physiological interaction. In addition, understanding this interaction is competitive or synergistic with regards to PD-1 binding and functional stimulation will be critical for biological understanding. The novel interaction of PD-L1 and PD-L2 warrants further investigations, which could enrich our understanding of the PD-1—PD-L1/PD-L2 inhibitory checkpoint pathway, and may provide new avenues for developing immunotherapy.

## Methods

### Magneto-nanosensor chip

The magneto-nanosensor chip has a dimension of 10 × 12 mm and an 8 × 8 array of sensors on the chip. Each sensor has a GMR spin valve structure of the type IrMn (8)/CoFe (2)/Ru (0.8)/CoFe (2)/Cu (2.3)/CoFe (4.5), where all numbers in parentheses are in nanometres. The area of each sensor is 100 × 100 μm, and the pitch from one sensor to the adjacent sensor is 300 μm. The sensors are connected to 300-nm-thick Ta/Au/Ta peripheral electrical pads in the manner of a grid network to be further connected to the reader station. To protect the sensors from corrosion and breakdown, a 30-nm-thick oxide layer was deposited on top of the sensors and a 300-nm-thick oxide layer was deposited on the rest of the sensor chip area^39^. The magnetization of free layer (4.5-nm-thick CoFe) is aligned perpendicular to that of the reference layer (CoFe layer underneath the copper layer) to use the most sensitive region of the magnetoresistance transfer curve, and can be rotated by stray field from MNPs. The relative orientation of magnetization in two layers determines spin-dependent scattering of electrons that pass through the sensor, which results in changes in magnetoresistance of the sensor.

In the reader station, an AC external magnetic field is applied to the chip by a Helmholtz coil at a frequency of 210 Hz, and the magnitude of the field is set to have a magnetoresistive ratio of 4%. A voltage of 0.4 V is applied to the sensor at a frequency of 540 Hz via the electrical pads to employ double modulation scheme^40^. The signals obtained from the sensors are further analyzed using fast Fourier transform to decouple the changes due to the MNPs from the entire signals.

### Microfluidic chip

The microfluidic chip used in our experiment has two layers of structure bonded via thermal diffusion process. The bottom layer has four channels interfaced with the magneto-nanosensor chip, and is connected to the top layer by vertical vias^41^. The channels were designed to have a width of 200 μm and a height of 50 μm, and moulds were fabricated using standard multilayer soft lithography with SU-8 photoresist on 4 inch silicon wafers. A mixture of 20 parts of base (RTV615A from Momentive) and 1 part of crosslinker (RTV615B from Momentive) was spin-coated on the mould of the bottom layer at speed of 1,300 r.p.m. to have a thickness of 100 μm. Then, the mould was baked using an oven at 80 °C for 40 min. The top layer has all routes for reagents and holes for tubing connection. Overall, 65 g of a mixture of five parts of base and one part of crosslinker was added to the mould of the top layer in a dish made of aluminium foil and baked at 80 °C for 1 h. After baking, the mould was detached, and punch holes were made for the tubing connectors. The top layer was then cut into chips of dimension 10 × 20 mm. Each chip was aligned on the top of the bottom layer and the whole assembly was baked together at 80 °C for 40 min. After baking, the bottom layer was cut along the edge of the chip, and the whole chips were detached from the mould.

To interface the microfluidic chip with the magneto-nanosensor chip, a cartridge provided a pressure seal between the microfluidic chip and the magneto-nanosensor chip. All reagents were delivered to the channels via tubing connected to a multi-channel syringe pump. The syringes and tubing were filled with degassed PBS with 0.5% BSA before the loading of reagents into the tubing. A total of 4% poloxamer 188 (Pluronic F-68) solution was injected into the channels and incubated for 15 min before every kinetic assay.

### Immobilization of proteins

The sensor surface was cleaned using acetone, methanol and isopropanol, and a temporary reaction well was installed on the chip to hold chemicals in the subsequent steps. The chip was further cleaned using oxygen plasma for 3 min before starting surface chemistry. First, the chip was treated with 1% poly(allylamine hydrochloride) for 5 min at room temperature, and rinsed with distilled water (10977-023 from Life Technologies). The chip was then baked using a hot plate at 120 °C for 1 h, followed by treatment with 2% poly(ethylene-alt-maleic anhydride) for 5 min^20^. Then, the chips were rinsed with distilled water, and a 1:1 mixture of *N*-hydroxysuccinimide (NHS) and 1-ethyl-3-(3-dimethylaminopropyl)carbodiimide hydrochloride (EDC) in distilled water was incubated with the chip for 1 h at room temperature. The chip was again rinsed with distilled water and fully dried. Each type of protein of interest was spotted on different sensors using a non-contact arrayer (sciFLEXARRAYER from Scienion). Each channel has the same configuration of sensors with the same kinds of proteins as shown in Supplementary Fig. 2. The chip was placed in a humid chamber and incubated overnight at 4 °C.

### High salt and pH varying experiment

Subclasses of murine IgG (IgG1, IgG2a, IgG2b and IgG3), IgM, biotinylated BSA and BSA were immobilized on different sensors in each channel. After overnight incubation at 4 °C, the chip was rinsed with washing buffer (PBS with 0.1% BSA and 0.05% Tween-20), and incubated with 1% BSA for 1 h. The chip was then assembled with a microfluidic chip, and the channels were filled with PBS pH 7.4 with 0.5% BSA. After obtaining the baseline signals, the syringe pumps (NE-1800 from New Era Pump Systems) were activated to flow four different buffer solutions containing MNPs coated with protein A from *S. aureus* and streptavidin, respectively, into each channel. For the buffer solutions, 90 μl of each buffer solution (PBS pH 7.4 from Life Technologies, Borate pH 8.5 from GE healthcare, acetate pH 5.5 from GE healthcare and 2 × SSC from Sigma-Aldrich) was mixed with 5 μl of protein A-coated MNPs (130-071-001 from Miltenyi Biotec) and 5 μl of streptavidin-coated MNPs (130-048-101 from Miltenyi Biotec).

### Conjugation of MNPs

To generate MNP complexes for kinetic assays, proteins of interest were conjugated with commercially available MNPs coated with protein A from *S. aureus* (130-071-001 from Miltenyi Biotec). The MNPs consist of multiple superparamagnetic cores embedded in a matrix of dextran, with a hydrodynamic diameter of 46 nm^18^. Since the proteins were tagged with human IgG1, they can bind to protein A on the MNPs via interaction between protein A and Fc-region of IgG1. Overall, 90 μl of proteins of interest at 50 μg ml^−1^ was mixed with 10 μl of protein A-coated MNPs, and incubated for 1 h at room temperature. After the incubation, the MNPs conjugated with the proteins were separated from the mixture using a magnetic separation unit (130-042-602 from Miltenyi Biotec). Briefly, the mixture was added to a column placed under a strong magnetic field, and the MNPs were trapped inside the column. After washing away unbound proteins, the external magnetic field was removed and the MNPs were eluted with PBS containing 0.1% BSA and 0.05% Tween-20. The concentration of the eluted solution was measured with a microplate reader. Overall, 100 μl of eluted MNPs were transferred to a well of a microplate. A known sample containing 1 nM of MNPs was diluted to be 0.9, 0.8, 0.6, 0.4, 0.2 and 0.1 nM, respectively, and duplicates of each were added to different wells of the plate. Absorbance at 425 nm was measured for all wells, and the concentration of eluted MNPs was calculated using signals from known samples.

### Kinetic assay

After protein immobilization on the sensors, the chip was rinsed with the washing buffer and incubated with 1% BSA for 1 h. Then, the chip was rinsed and incubated with soluble protein A (P7837 from Sigma-Aldrich) at 1 mg ml^−1^ for 30 min. The chip was again rinsed with the washing buffer, and the temporary reaction well was removed. A microfluidic chip was then integrated with the chip using the clamping device, and the channels were treated with 4% poloxamer 188 for 15 min. The conjugated MNPs with the proteins were diluted to four different concentrations (100, 75, 50 and 25% of the concentration after separation). Four different solutions at different concentrations were loaded into the tubing connected to the syringe pumps, and flowed into each channel at a flow rate of 1 μl min^−1^ after obtaining the baseline signals. The signals from 64 sensors were recorded every 5.5 s.

### Curve fitting algorithm

The method of non-linear least squares was used to fit the binding curves obtained from the kinetic assays with equation (2) based on a Langmuir isotherm model. The reference signals were subtracted from all binding signals, and 80 data points after the onset of binding were used for the analyses. From the curve fitting, the constant, *k*_obs_, was calculated and plotted versus the corresponding concentration of the conjugated MNPs. A linear regression scheme was used to fit four data points from four channels with a line. If multiple experiments were performed, the collection of all data points was used for the linear regression. The slope of the line is *k*_on_ and the *y*-intercept is *k*_off_ of the interaction because the relationship between these parameters is 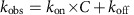, where *C* is the concentration of conjugated MNPs.

### Surface plasmon resonance

Surface plasmon resonance analysis was carried out using a Biacore 3000 instrument. Proteins were immobilized, using the NHS/EDC method, on a CM5 chip at the indicated RU (resonance unit) levels. Analytes were run at 20 μl min^-1^ in HBS-P buffer (0.01 M HEPES, pH 7.4, 0.15 M NaCl, 0.005% Tween-20) at the indicated concentrations for 3 min. Chip regeneration was carried out by injecting glycine pH 3.0 for 30 s.

### Cell-based experiment

For each independent screen, COS-7 cells (2,500 cells per well, 384-well plate) were reverse transfected with 60 ng of cDNA, representing most full-length human single-transmembrane proteins (Genentech Proprietary Library), one clone per well, using Lipofectamine LTX-Plus reagent at 3.2:1 Lipofectamine LTX-Plus:DNA ratio. Cells were incubated for 48 h at 37 °C, 5% CO_2_ before the assay. After 48 h, the spent media was washed with 1 × PBS and COS-7 cells were blocked using 1% BSA at 4 °C for 30 min. Post blocking, the cells were washed and incubated with probes of interest (PD-L1-Fc and PD-L1-Fc-biotin complexed with streptavidin-APC) at 4 °C for 45 min to allow baits to bind specifically to proteins expressed on cell surface. Following incubation with probe proteins, the cells were washed and fixed for 20 min at room temperature using 4% paraformaldehyde. For the PD-L1-Fc screen, a secondary antibody F(ab′)_2_ fragment goat anti-human IgG (H+L) conjugated with allophycocyanin (APC) was used to stain and detect any interacting partners. For the screen using PD-L1-Fc-biotin conjugated with streptavidin-APC, detection of interacting partners was done directly after washing the fixative. Fluorescence images were acquired using an IN Cell Analyzer 6000 (GE Healthcare). Image analysis was performed using IN Cell Developer Toolbox version 1.9.3 software. The IN Cell Developer Toolbox software was used to calculate the total intensity values from each well.

### Data availability

The authors declare that the data supporting the findings of this study are available within the article and its Supplementary Information, or from the corresponding author upon request.

## Additional information

**How to cite this article:** Lee, J.-R. *et al*. Magneto-nanosensor platform for probing low-affinity protein–protein interactions and identification of a low-affinity PD-L1/PD-L2 interaction. *Nat. Commun.* 7:12220 doi: 10.1038/ncomms12220 (2016).

## Supplementary Material

Supplementary Information Supplementary Figures 1-6, Supplementary Table 1 and Supplementary Note 1

## Figures and Tables

**Table 1:** Dissociation constants (*K*_D_) of human PD-1, PD-L1, PD-L2 and B7-1, measured by magneto-nanosensor platform.

Prey (flowed)	Bait (immobilized)	Magneto-nanosensors (μM)	Literature (μM)^14^
PD-1	PD-L1	5.2	8.2
PD-L1	PD-1	4.9	7.5
PD-1	PD-L2	4.7	2.3
PD-L2	PD-1	6.4	2.2
PD-L1	B7-1	9.1	35.4
B7-1	PD-L1	9.8	18.8
PD-L1	PD-L2	10.7	N/A
PD-L2	PD-L1	9.1	N/A
