# Supplementary material for: Magneto-nanosensor platform for probing low-affinity protein–protein interactions and identification of a low-affinity PD-L1/PD-L2 interaction
Source: Nat Commun. 2016 Jul 22;7:12220. doi: 10.1038/ncomms12220 (PMC4961847; doi:10.1038/ncomms12220)
Supplement: Supplementary Information — Supplementary Figures 1-6, Supplementary Table 1 and Supplementary Note 1 [file ncomms12220-s1.pdf]

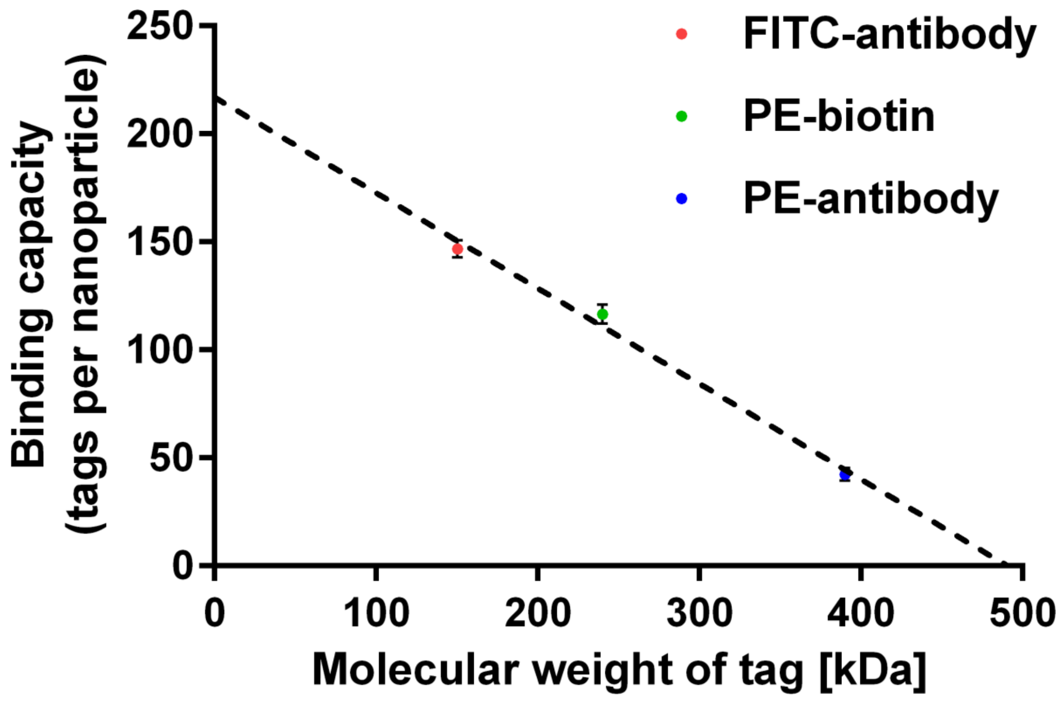

Supplementary Figure 1. Binding capacity of the nanoparticles. Protein A-coated magnetic nanoparticles were mixed with FITC-antibody (antibody conjugated with FITC) and PE-antibody (antibody conjugated with PE), while streptavidin-coated magnetic nanoparticles were mixed with PE-biotin. After washing unbound tags, the concentrations of tags and nanoparticles were determined, and the number of tags on the particles was calculated. The error bars represent 3 standard deviations of 2 identical measurements.

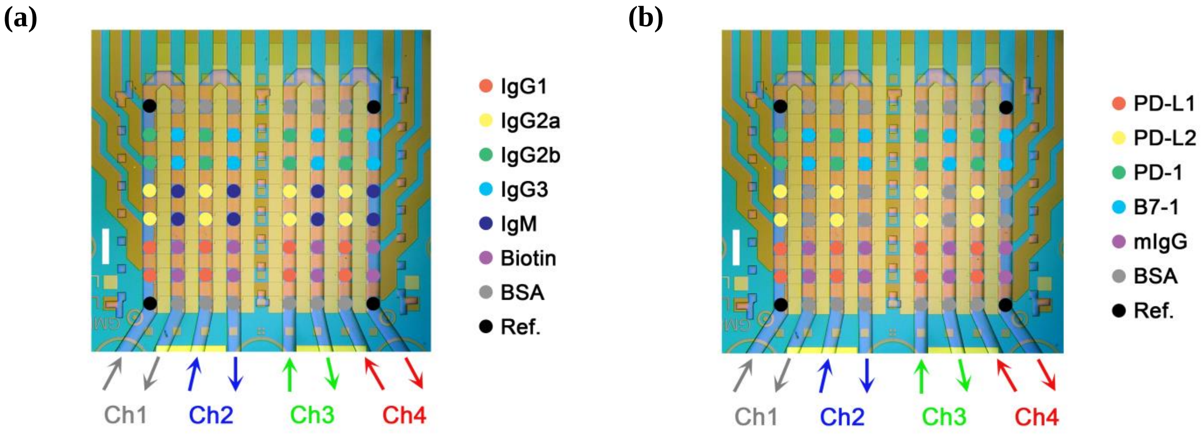

Supplementary Figure 2. Configurations of immobilization of bait proteins. (a) Configuration of bait protein immobilization on magneto-nanosensor chip for pH and salinity sensitivity test: isotypes of mouse IgG, mouse IgM, biotin (biotinylated BSA), and BSA. The reference sensors labeled as Ref. (in black) are the sensors with a thick passivation layer as electrical negative controls. (b) Configuration of bait protein immobilization on magneto-nanosensor chip for interactions of PD-1 system: PD-1, PD-L1, PD-L2, B7-1, mouse IgG, and BSA. The scale bar is 500 µm.

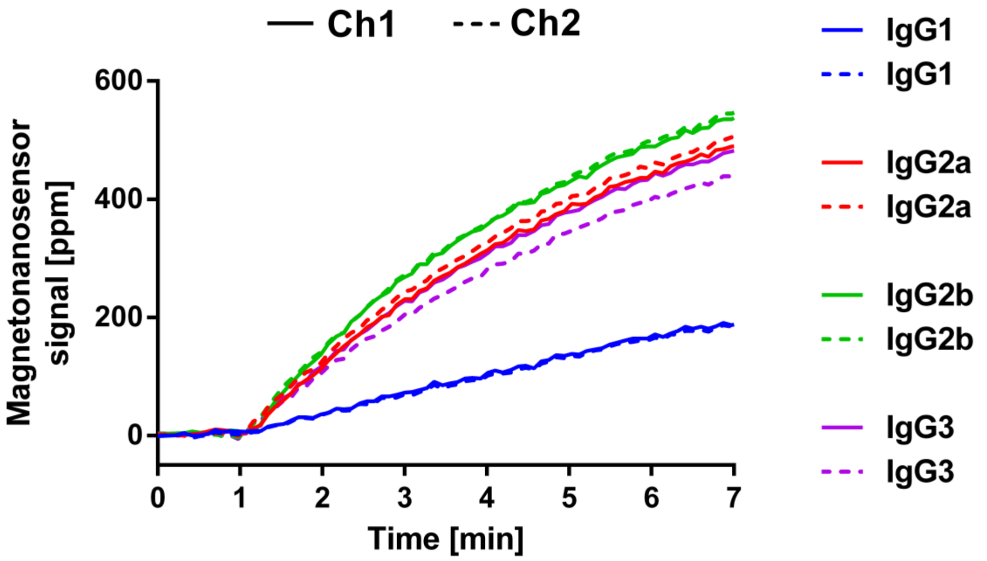

Supplementary Figure 3. Reproducibility of assays. The same concentration of protein A-coated particles was flowed into two channels with identical immobilization patterns of mouse IgG isotypes (IgG1, IgG2a, IgG2b, and IgG3). Solid curves were obtained in the first channel, and dashed curves were measured in the second channel. Each curve was the binding signal from a single sensor. The signals from two channels were post-synchronized across the channels.

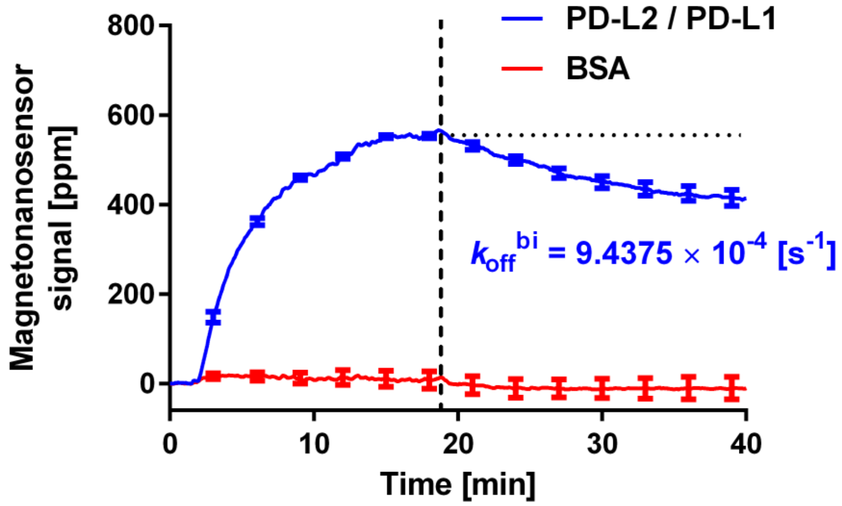

Supplementary Figure 4. Direct measurement of dissociation rate of the bivalent interaction between PD-L2 and PD-L1. PD-L1 was immobilized on the sensors, and complexes with PD-L2 were added. While we started to flow a washing buffer solution (PBS pH 7.4 with 0.1 % BSA) at ~ 19 min as indicated by the dashed line, we monitored unbinding signals over 20 min. The dissociation signals were used to calculate the dissociation rate of the interaction. Error bars represent standard deviations of 4 identical sensor signals.

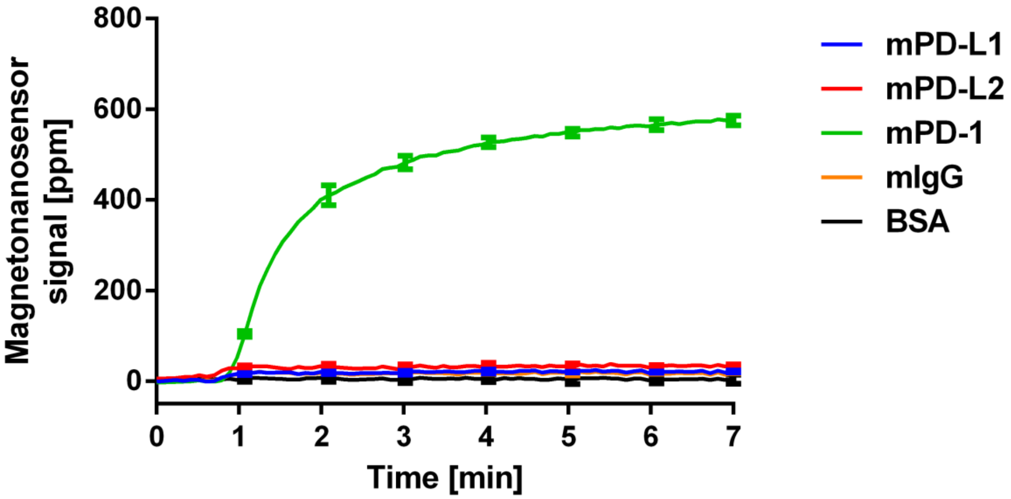

Supplementary Figure 5. Interactions between murine PD-1 systems. The complexes with murine PD-L1 were flowed over the sensors coated with murine PD-L1 (blue), PD-L2 (red), and PD-1 (green). Bindings of murine PD-L1 to murine PD-1 were observed, but not to murine PD-L2. The error bars are standard deviations of signals from two identical sensors.

(a)

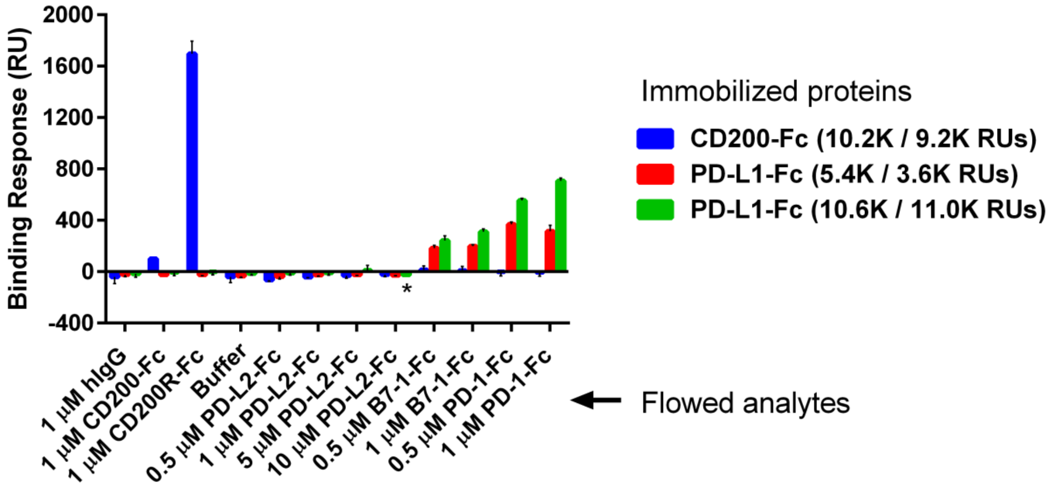

(b)

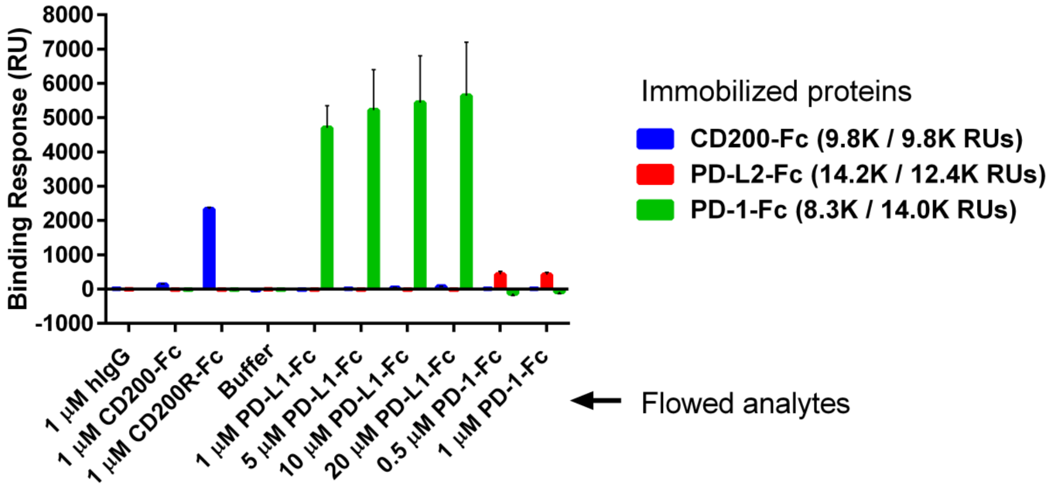

Supplementary Figure 6. PD-L1 and PD-L2 do not interact via SPR. (a) SPR binding responses for PD-L2-Fc analyte flowed over immobilized PD-L1-Fc at high (10.6K and 11.0K RUs) and low (5.4K and 3.6K RUs) immobilization levels. The error bars represent standard deviations of two independent sets of experiments (No error bar is shown for the signal indicated by an asterisk due to high consumption of reagents). Each immobilization level in parentheses was used for each set of experiments. RU responses were recorded shortly after the end of the analyte injection. CD200, CD200R, and human whole IgG (hIgG) were used as negative controls. (b) SPR binding responses for PD-L1-Fc analyte flowed over immobilized PD-L2-Fc (14.2K and 12.4K RUs) and PD-1-Fc (8.3K and 14.0K RUs).

Supplementary Table 1. Estimated dissociation constants (K_D) with monovalent or trivalent binding modes

Prey (Flowed)	Bait (Immobilized)	Monovalent	Trivalent
PD-1	PD-L1	27.0 pM	0.30 mM
PD-L1	PD-1	24.0 pM	0.29 mM
PD-1	PD-L2	22.1 pM	0.28 mM
PD-L2	PD-1	40.1 pM	0.34 mM
PD-L1	B7-1	82.8 pM	0.44 mM
B7-1	PD-L1	96.0 pM	0.46 mM

Supplementary Note 1. Flow regime in the microfluidic channel

The bulk velocity of flow in the microfluidic channel at 1 µL min^{-1} is 1.67 mm s^{-1} (cross-sectional area of the channel is 200 µm × 50 µm).

$$u = \frac{1\ \mu L\ min^{-1}}{200\ \mu m \times 50\ \mu m} = 1.67\ mm\ s^{-1}$$

If we assume that kinetic viscosity of that liquid flowed into the channel is similar to that of water at 20 °C, it can be 1.004 × 10^6 m^2 s^{-1}. The Reynolds number of the flow is then 3.19 × 10^{-5}, which means the flow is laminar.

$$Re = \frac{u \cdot L}{\nu} = \frac{1.67\ mm\ s^{-1} \times 50\ \mu m}{1.004 \times 10^6\ m^2\ s^{-1}} = 3.19 \times 10^{-5} \ll 1$$

In the laminar regime, turbulent mixing of nanoparticles does not occur. Thus, mass transport and homogenization rely on diffusion and convection. If diffusion is a limiting factor for the mass transport, binding events are slowed down. The Péclet number can determine which mass transport mechanism is dominant.

$$D = \frac{k_B T}{6\pi\eta r} = \frac{1.38 \times 10^{-23}\ J\ K^{-1} \times 300\ K}{6\pi \times 1.002 \times 10^{-3}\ N\ s\ m^{-2} \times 25 \times 10^{-9}\ m} = 8.77 \times 10^{-12}\ m^2\ s^{-1}$$

$$Pe = \frac{Q}{D \cdot W} = \frac{1\ \mu L\ min^{-1}}{8.77 \times 10^{-12}\ m^2\ s^{-1} \times 200\ \mu m} = 9502 \gg 1$$

The Péclet number of this flow is 9502, assuming nanoparticles with a diameter of 50 nm diffusing through water at 20 °C, which means the flow is much faster than diffusion so nanoparticles can be replenished by the flow.
